# A regulatory variant of *CHRM3* is associated with cannabis-induced hallucinations in European Americans

**DOI:** 10.1038/s41398-019-0639-7

**Published:** 2019-11-18

**Authors:** Zhongshan Cheng, Chureerat Phokaew, Yi-Ling Chou, Dongbing Lai, Jacquelyn L. Meyers, Arpana Agrawal, Lindsay A. Farrer, Henry R. Kranzler, Joel Gelernter

**Affiliations:** 1 Division of Human Genetics, Department of Psychiatry, VA CT Healthcare Center, Yale University School of Medicine, New Haven, CT USA; 2 Department of Psychiatry, Washington University School of Medicine, St. Louis, MI USA; 3 Department of Medical and Molecular Genetics, Indiana University School of Medicine, Indianapolis, IN USA; 4 Department of Psychiatry, State University of New York Downstate Medical Center, Brooklyn, NY USA; 5 Departments of Neurology, Ophthalmology, Genetics & Genomics, Epidemiology and Biostatistics, Boston University Schools of Medicine and Public Health, Boston, MA USA; 6 Department of Psychiatry, Center for Studies of Addiction and Crescenz Veterans Affairs Medical Center, University of Pennsylvania Perelman School of Medicine, Philadelphia, PA USA; 7 Departments of Genetics and Neuroscience, Yale University School of Medicine, New Haven, CT USA

**Keywords:** Predictive markers, Medical genetics

## Abstract

Cannabis, the most widely used illicit drug, can induce hallucinations. Our understanding of the biology of cannabis-induced hallucinations (Ca-HL) is limited. We used the Semi-Structured Assessment for Drug Dependence and Alcoholism (SSADDA) to identify cannabis-induced hallucinations (Ca-HL) among long-term cannabis users (used cannabis ≥1 year and ≥100 times). A genome-wide association study (GWAS) was conducted by analyzing European Americans (EAs) and African Americans (AAs) in Yale-Penn 1 and 2 cohorts individually, then meta-analyzing the two cohorts within population. In the meta-analysis of Yale-Penn EAs (*n* = 1917), one genome-wide significant (GWS) signal emerged at the *CHRM3* locus, represented by rs115455482 (*P* = 1.66 × 10^−10^), rs74722579 (*P* = 2.81 × 10^−9^), and rs1938228 (*P* = 1.57 × 10^−8^); signals were GWS in Yale-Penn 1 EAs (*n* = 1092) and nominally significant in Yale-Penn 2 EAs (*n* = 825). Two SNPs, rs115455482 and rs74722579, were available from the Collaborative Study on the Genetics of Alcoholism data (COGA; 3630 long-term cannabis users). The signals did not replicate, but when meta-analyzing Yale-Penn and COGA EAs, the two SNPs’ association signals were increased (meta-*P*-values 1.32 × 10^−10^ and 2.60 × 10^−9^, respectively; *n* = 4291). There were no significant findings in AAs, but in the AA meta-analysis (*n* = 3624), nominal significance was seen for rs74722579. The rs115455482*T risk allele was associated with lower *CHRM3* expression in the thalamus. *CHRM3* was co-expressed with three psychosis risk genes (*GABAG2*, *CHRNA4*, and *HRH3*) in the thalamus and other human brain tissues and mouse GABAergic neurons. This work provides strong evidence for the association of *CHRM3* with Ca-HL and provides insight into the potential involvement of thalamus for this trait.

## Introduction

Cannabis is the most widely used illicit drug. It has acute and chronic effects on physical and mental health; adverse effects include rapid heartbeat, disorientation, lack of physical coordination, panic attacks or anxiety, depression or sleepiness, deterioration in cognitive function, and brain abnormalities after long-term use^1–3^. The pharmacological effects of cannabis are due primarily to tetrahydrocannabinol (THC), which mimics the activity of endocannabinoids such as anandamide. Both THC and endocannabinoids efficiently bind to the G-protein-coupled cannabinoid receptor, CB1, in the brain and transiently inhibit the release of either the inhibitory neurotransmitter γ-aminobutyric acid (GABA) or the excitatory transmitter glutamate^4^. Although there is another well-characterized cannabinoid G-protein-coupled receptor, CB2, only CB1 receptors are abundantly expressed in the brain, where they are localized specifically on axons and axon terminals. These effects are largely responsible for the psychoactive effects of cannabis, which include potentially therapeutic ones (e.g., analgesia) and reinforcing effects (e.g., relaxation, hallucination, or altered perception)^3,5^. Our understanding of the biology of cannabis-induced hallucinations (Ca-HL) remains limited.

Genome-wide association study (GWAS) is a useful strategy to study the genetics and ultimately the biology of hallucinations. Here we carried out the first GWAS of Ca-HL among European American (EA) (total *N* = 4291) and African American (AA) (total *N* = 3624) long-term cannabis users. We identified one genome-wide significant (GWS) signal close to the gene cholinergic receptor muscarinic 3 (*CHRM3*), predisposing to Ca-HL in EAs, with the finding nominally replicated in AAs.

## Materials and methods

### Subjects, genotyping, and imputation

Subjects were selected from among previously described samples, Yale-Penn 1 (*n* = 5540) and 2 (*n* = 3675)^6^, which were recruited from five eastern US sites to participate in studies of the genetics of drug (opioid or cocaine) or alcohol dependence^6^. All participants were given written informed consent that was approved by the institutional review board at each recruiting site. Certificates of confidentiality were provided by the National Institute on Drug Abuse and the National Institute on Alcohol Abuse and Alcoholism. Yale-Penn 1 samples were genotyped on the Illumina (San Diego, CA, USA) HumanOmni1-Quad v1.0 microarray. A total of 1,140,419 single-nucleotide polymorphisms (SNPs) were genotyped in Yale-Penn 1. Samples for Yale-Penn 2 were genotyped with the Illumina HumanCore Exome array, which includes a total of 550,601 SNPs, including 268,631 exonic SNPs and 281,970 tagging SNPs. Quality control (QC) for microarrays in each cohort was carried out using PLINK1.9^7^ based on the following criteria: (1) individual genotype missing rate < 2%, (2) SNP genotype missing rate < 2%, (3) Hardy–Weinberg *P* > 1 × 10^−6^, and (4) minor allele frequency (MAF) > 3%. After QC, samples from Yale-Penn 1 and 2 were subjected to ancestry analysis by comparison with the 1000 Genomes Project phase 1 reference panel^8^. Eigensoft^9^ was used for principal components (PCs) analysis with the first ten PC scores serving to differentiate EAs and AAs through K-means clustering^10^. For each Yale-Penn cohort, SNPs from EAs and AAs were imputed together using Minimac3 implemented in the Michigan Imputation Server^11^ with the 1000 Genomes phase 3 reference panel. We transformed dosage data into best-estimate genotypes using PLINK1.9, retaining high-quality genotyping data by filtering imputed data with genotype imputation probability (GP) ≥ 0.9; the resulting genotype data were transformed into plink binary format data, which can be used directly in association tests with the GWAS software, Genome-wide Efficient Mixed Model Association (GEMMA)^12^. After retaining genotypes with GP ≥ 0.9, individual genotyping missing rate < 5%, MAF > 3%, and missing call frequency < 5%, there were 8,200,853 and 5,916,265 remaining variants for Yale-Penn 1 and 2 EAs, respectively, and 14,134,502 and 10,346,266 variants for Yale-Penn 1 and 2 AAs, respectively.

Apart from the above Yale-Penn samples used in our discovery GWAS, an independent cohort, the Collaborative Study on the Genetics of Alcoholism data (COGA; see Supplementary Information for detailed description of COGA) was assigned as the replication cohort for the top hits that emerged from Yale-Penn samples.

### Definition of Ca-HL

Ca-HL was defined using similar questions in the Semi-Structured Assessment for Drug Dependence and Alcoholism (SSADDA)^13^ (for Yale-Penn) and the Semi-Structured Assessment for the Genetics of Alcoholism (SSAGA)^14^ (for COGA). For the SSADDA, this question is, “Has your use of [marijuana] ever caused you emotional or psychological problems like: Hearing, seeing, or smelling things that weren’t really there?” In the SSAGA, the question is “Because of your marijuana use, did you ever experience any of the following: Hearing, seeing or smelling things that weren’t really there?”. As in Yale-Penn samples >90% of participants with Ca-HL used cannabis ≥1 year and ≥100 times, these two criteria were used to define comparison subjects (controls) who were long-term cannabis users without Ca-HL. The same criteria were used to select samples from COGA. The sample sizes of our GWAS were not predesigned for ensuring adequate power to detect a pre-specific effect size.

### Ca-HL sample description

For Ca-HL samples in Yale-Penn cohorts, there were 1092 Yale-Penn 1 EAs (cases = 51 and controls = 1041), 825 Yale-Penn 2 EAs (cases = 40 and controls = 785), 1610 Yale-Penn 1 AAs (cases = 149 and controls = 1461), and 758 Yale-Penn 2 AAs (cases = 71 and controls = 687). Meanwhile, for replication samples from COGA, 2374 EAs (cases = 256 and controls = 2118) and 1256 (cases = 142 and controls = 1114) AAs had genotypic data and reported long-term use of cannabis.

### GWAS analysis

#### GWAS software

GEMMA^12^ was used separately in each Yale-Penn subgroup of EAs and AAs, with adjustment for sex, age, body mass index (BMI), the first three PCs of ancestry, and the degree of relatedness among subjects. BMI was used as covariant because of the consideration that it could be a potential confounding factor affecting Ca-HL. GEMMA uses linear mixed model to determine the association between SNP and phenotype; it can account for relatedness among participants and can control for population stratification and other confounding factors^12^. We used GEMMA because it allowed us to account for ancestry. A correction for inflation was not necessary, because the inflation was low (all *λ* < 1.1) in our GWAS. For summary statistics from GEMMA, the inverse variance method implemented in PLINK1.9 was used to generate fixed-effects meta-analysis *P*-values (meta-*P*) for all variants by matching their chromosomal positions and two alleles among the GWAS datasets from EAs and AAs separately. We used a GWS threshold of *P* < 5.0 × 10^−8^. GWAS summary statistics data for Yale-Penn samples are freely available upon request.

For the two GWS variants that emerged in the meta-analysis of Yale-Penn cohorts, we used logistic regression via generalized estimating equation implemented in the R package GWAF^15^, to correlate the two GWS variants’ genotypes from COGA with Ca-HL (defined exactly as for Yale-Penn) and adjust for sex, age, the first three PCs of ancestry, and array types. As BMI was not available in COGA, it was not included as a covariate in the analysis. The association results for these two GWS variants were combined with the summary statistics of Yale-Penn 1 and 2, and COGA in the final meta-analysis using PLINK1.9.

### Correlation between rs115455482 genotype and *CHRM3* expression

To facilitate the biological interpretation of our top association signal, we explored the potential relationship between rs115455482 and *CHRM3* expression across ten brain tissues. We associated rs115455482 genotype with *CHRM3* expression in Braineac^16^, where genotype and gene expression data of ten brain tissues, including occipital cortex (OCTX), frontal cortex (FCTX), temporal cortex, hippocampus (HIPP), intralobular white matter, cerebellar cortex (CRBL), thalamus (THAL), putamen (PUTM), substantia nigra (SNIG), and medulla (inferior olivary nucleus, MEDU), were obtained from 134 healthy human brains from the UK Biobank^17^.

### *CHRM3* co-expression analysis and disease gene-enrichment analysis

Gene co-expression information can be used to evaluate a gene’s function and identify related pathways. To accomplish this for *CHRM3* in Ca-HL, a novel bioinformatics pipeline was created to search for genes co-expressed with *CHRM3* in an unbiased way genomewide. First, *CHRM3* co-expressed genes were identified with COXPRESdb^18^, a database that provides gene co-expression relationships for animal species, including human, mouse, rat, and others. Next, the top 100 *CHRM3* co-expressed genes, as well as *CHRM3* itself (total 101 genes), were subjected to enrichment analysis of disease-associated genes using the 2013 version WEB-based GEne SeT AnaLysis Toolkit (WebGestalt)^19^ with default settings of a minimum number of four genes (out of 101) required for a gene category to be included in the enrichment analysis. Enrichment analysis method “Disease Association Analysis” of WebGestalt was used to test the enrichment of disease-associated genes among these 101 genes. Finally, *CHRM3* co-expressed genes that emerged in the top ten enriched gene categories of disease-associated genes were validated for co-expression with *CHRM3* using ten different types of human healthy brain tissues (downloaded from Braineac database)^17^ and the homologous gene in mouse, *Chrm3*, using six types of cortical GABAergic neurons from mouse FCTX (gene expression omnibus accession number GSE92522)^20^. The ten brain tissues (total samples = 1340) are the same as these listed above. In addition, we considered six types of mouse single neurons (total samples = 584): martinotti cells, interneuron selective cells, cholecystokinin expressing (CCK)-basket cells, parvalbumin expressing (PV) basket cells, chandelier cells upper layer (CHC1) and deep layer (CHC2) of mouse brain, and long projecting cells. All of the cells were isolated from mouse FCTX. The correlation between *CHRM3* and its co-expressed genes emerged in the enrichment of disease-associated genes was analyzed separately among ten brain tissues for Pearson’s correlation by using MATLAB (Statistics Toolbox Release 2015b, The MathWorks, Inc., Natick, Massachusetts, USA). The same correlation analysis was performed for the mouse homologous *Chrm3* among six types of single neurons. The adjusted *P*-value for significance was 0.05/(10*24) = 2 × 10^−4^ for the co-expression analysis with 10 brain tissues for 24 genes, and in the co-expression analysis of 6 mouse GABAergic neurons for 14 genes, the adjusted significance *P*-value was 0.05/(6*14) = 5 × 10^−4^.

## Results

**Fig. 1:**
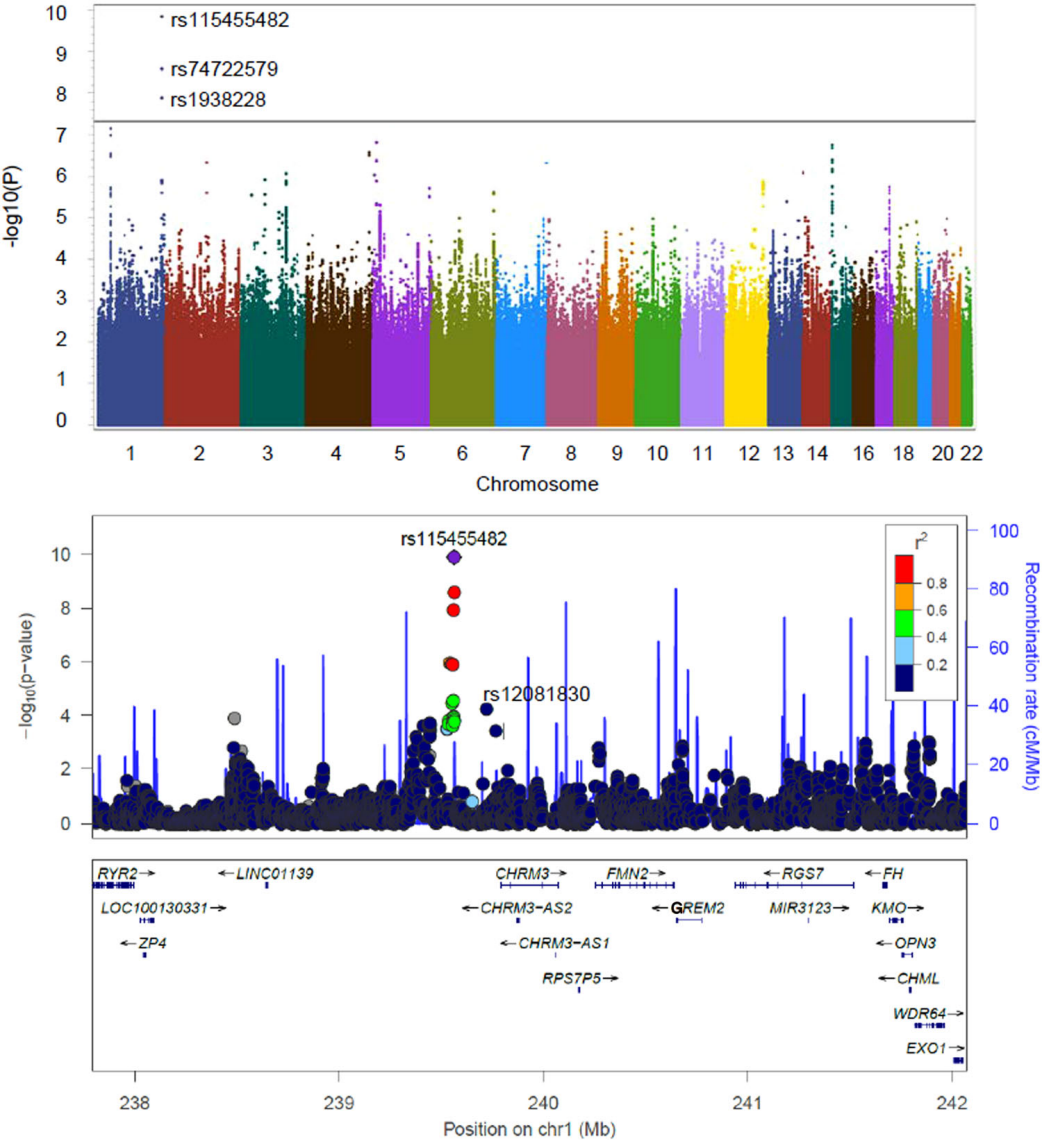
Manhattan plots showing genome-wide association signals of cannabis-induced hallucinations in long-term cannabis-exposed European Americans (EAs) by meta-analysis. **a** Manhattan plot with three significant variants, rs115455482, rs74722579, and rs1938228, on chromosome 1, in a meta-analysis of two EA cohorts. The three variants are in high linkage disequilibrium (all *r*^2^ ≥ 0.95) in EAs. The line in the plot represents the genome-wide significance cutoff (5 × 10^−8^). **b** Regional Manhattan plot demonstrates rs115455482 is close to gene *CHRM3* (and regulates *CHRM3* expression; see Fig. 2 and text). According to a published schizophrenia genome-wide association study^36^, these top three SNPs associated with cannabis-induced hallucinations were not significantly associated with schizophrenia (all associations *P* ~ 0.25); one SNP rs12081830, associated with cannabis-induced hallucination (*P* = 8.5 × 10^−5^), was nominally associated with schizophrenia (*P* = 4.3 × 10^−2^). rs115455482 and rs12081830 are not highly linked with each other (*r*^2^ < 0.2 in EAs). The light blue line and right Y-axis show the observed recombination rate in the 1000 Genomes Project European samples (EUR, hg19). The SNPs are colored in accordance to *r*^2^ with rs115455482.

In the discovery GWAS, we meta-analyzed Yale-Penn 1 and 2 EAs and AAs separately by population (Fig. 1). In the meta-analysis of Yale-Penn EAs, three GWS SNPs representing one signal were identified (see GWAS results in Fig. 1 for EAs): rs115455482, rs74722579, and rs1938228, which are highly correlated (*r*^2^ ≥ 0.95, MAF ~ 0.06 in EAs when cases and controls were considered together; Table 1 and Supplementary Table S1). The association *P*-values were 1.66 × 10^−10^, 2.81 × 10^−9^, and 1.57 × 10^−8^ for rs115455482, rs74722579, and rs1938228, respectively; the leading SNP rs115455482 is ~230 kb upstream of transcript NM_000740 of *CHRM3* (muscarinic acetylcholine receptor (AChR) M3). There were no GWS SNPs identified in AAs (Supplementary Figs. 1 and 2 for AAs). Low inflation was observed in the meta-analysis of Yale-Penn 1 and 2 for EAs (*λ* = 1.06; Supplementary Fig. 3) and AAs (*λ* = 1.02; Supplementary Fig. 2).

**Table 1:** Meta-analysis of rs115455482 and rs74722579 in European American (EA) and African American (AA) long-term cannabis users.

Pop	Cohort^a^	Hallucination control no.	Hallucination case no.	SNP^b^	Allele	MAF^c^		Effect *β* (SE)^d^	*P*
						Case	Control		
EA	Yale-Penn 1	1041	51	rs115455482	T	0.167	0.040	0.13 (0.03)	4.93 × 10^−9^
				rs74722579	C	0.177	0.048	0.12 (0.03)	1.66 × 10^−8^
	Yale-Penn 2	785	40	rs115455482	T	0.088	0.031	0.08 (0.02)	4.08 × 10^−3^
				rs74722579	C	0.088	0.036	0.07 (0.02)	3.15 × 10^−2^
	COGA^e^	2118	256	rs115455482	T	0.059	0.058	0.05 (0.21)	8.08 × 10^−1^
				rs74722579	C	0.063	0.064	0.02 (0.20)	9.34 × 10^−1^
	**Meta-analysis**	**3944**	**347**	**rs115455482**	**T**			**0.11**	**1.32** **×** **10**^**−10**^
				**rs74722579**	**C**			**0.10**	**2.60** **×** **10**^**−9**^
AA	Yale-Penn 1	1461	149	rs115455482	T	0.017	0.007	0.10 (0.06)	9.97 × 10^−2^
				rs74722579	C	0.074	0.048	0.05 (0.02)	5.27 × 10^−2^
	Yale-Penn 2	687	71	rs115455482	T	0.015	0.005	0.02 (0.01)	8.65 × 10^−1^
				rs74722579	C	-	-	-	-
	COGA	1114	142	rs115455482	T	0.011	0.013	−0.12 (0.61)	8.40 × 10^−1^
				rs74722579	C	0.103	0.088	0.19 (0.26)	4.50 × 10^−1^
	**Meta-analysis**	**3262**	**362**	**rs115455482**	**T**			**0.06**	**1.37** **×** **10**^**−1**^
				**rs74722579**	**C**			**0.05**	**4.58** **×** **10**^**−2**^
EA + AA	**Meta-analysis**	**7206**	**709**	**rs115455482**	**T**			**0.11**	**5.66** **×** **10**^**−11**^
				**rs74722579**	**C**			**0.08**	**1.60** **×** **10**^**−9**^

^a^Illumina microarray HumanOmni1-Quad v1.0 and HumanCore Exome array were used to genotype samples for Yale-Penn 1 and 2, respectively

^b^Rs115455482 is highly linked with rs74722579 in EA (*r*^2^ = 0.95) but not in AAs (*r*^2^ < 0.2). rs115455482 is a common variant in EAs but a rare variant in AAs, meanwhile rs74722579 is a common variant both in EAs and AAs

^c^Minor allele frequency

^d^Effect *β* and SE based on likelihood ratio test by using the software Genome-wide Efficient Mixed Model Association (GEMMA)

^e^COGA: Collaborative Study on the Genetics of Alcoholism

Note: The two variants are imputed with imputation score > 0.8 among all cohorts. Cannabis users who use cannabis ≥1 year and ≥100 times are defined as long-term cannabis users. Results for meta-analyses of rs115455482 and rs74722579 among AAs, EAs and AAs+EAs are highlighted in bold in the table

The independent COGA sample was used to attempt to replicate these top signals in the discovery GWAS. Two of these SNPs, rs115455482 and rs74722579, were available in the COGA data; neither of these were significantly associated with trait, although their *β*-effects were both in the same direction (0.05 and 0.02, respectively). Further meta-analysis of both Yale-Penn discovery EA samples with the COGA EA replication sample (Table 1) for these SNPs increased the statistical significance of the association signals (meta-*P*-values of 1.32 × 10^−10^ and 2.60 × 10^−9^ for rs115455482 and rs74722579, respectively). In addition, meta-analysis for rs74722579 (MAF ~ 0.10) including all available AA samples from Yale-Penn 1 and 2, and COGA (total cases = 362 and controls = 3404) yielded a nominally significant meta-*P*-value (meta-*P* = 4.58 × 10^−2^); the analysis for rs115455482 was not significant. For the latter variant, the minor allele is rare in AAs (MAF = 0.01, meta-*P* = 1.37 × 10^−1^) (Table 1). Taken together, the robust association signals for the two top SNPs in EAs and the nominal significance for rs74722579 in AAs (Fig. 1 and Table 1) support the validity of the GWAS results.

**Fig. 2:**
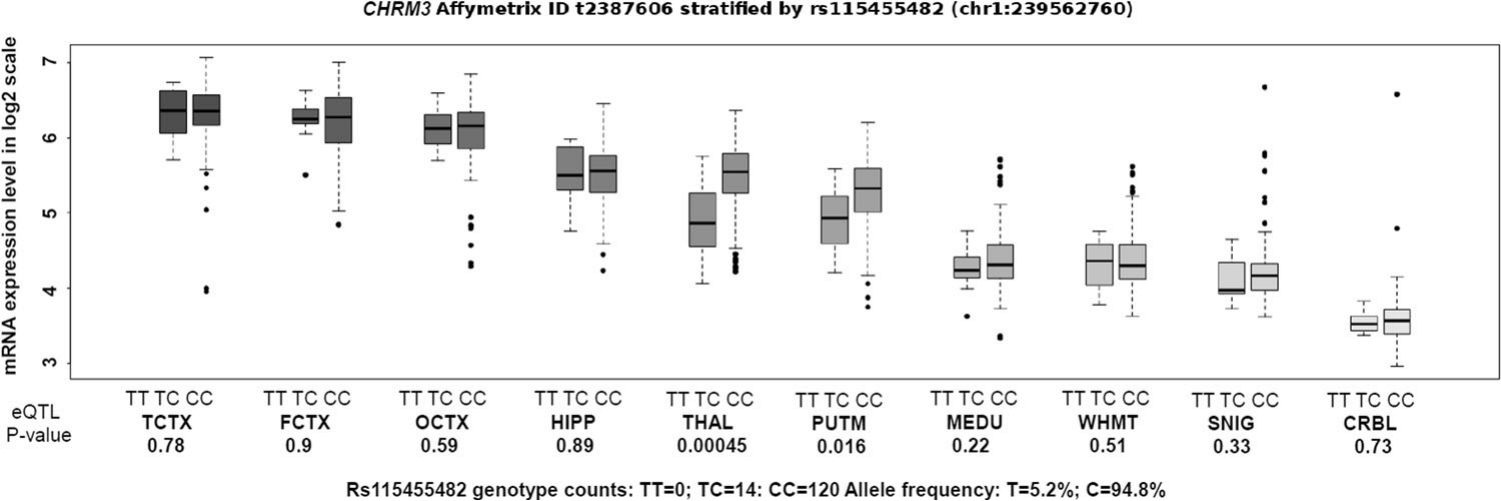
rs115455482 is a regulatory variant of *CHRM3*. The correlation between rs115455482 genotype and *CHRM3* expression across ten brain tissues in European samples. Line within each boxplot represents median and box region indicates the range from first quantile to third quantile. Up and down whiskers, as well as dots outside box region, represent maximum value, minimum value, and outliers, respectively. The boxplot was generated in Braineac (http://www.braineac.org/). The abbreviations for ten brain tissues are CRBL (cerebellar cortex), FCTX (frontal cortex), HIPP (hippocampus), MEDU (medulla specifically inferior olivary nucleus), OCTX (occipital cortex), PUTM (putamen), SNIG (substantia nigra), TCTX (temporal cortex), THAL (thalamus), and WHMT (intralobular white matter). Affymetrix ID t2387606 represents transcript IDs for *CHRM3* at mRNA level.

To evaluate a potential underlying mechanism for the association of rs115455482 genotype with Ca-HL, the regulatory relationship between rs115455482 and *CHRM3* expression was evaluated in the Braineac database. In expression quantitative trait locus (eQTL) analysis, *CHRM3* was variably expressed among ten brain tissues and differentially expressed among rs115455482*T risk allele carriers in the THAL (*P* = 4.5 × 10^−4^; adjusted *P* = 4.5 × 10^−3^ across ten brain tissues) and PUTM (*P* = 1.6 × 10^−2^; adjusted *P* = 1.6 × 10^−1^ across ten brain tissues) (Fig. 2). Rs115455482 risk genotype TC (TT homozygotes were too rare to be observed in this sample) was correlated with lower mRNA expression of *CHRM3* in THAL, with the same trend found in other tissues, including PUTM, OCTX, MEDU, SNIG, FCTX, HIPP, and CRBL.

To infer the potential biological function of *CHRM3*, we carried out co-expression analysis. We obtained the top 100 co-expressed genes (Supplementary Table S2) with respect to *CHRM3* by application of COXPRESdb^18^ and then performed enrichment analysis in disease-associated gene categories in WebGestalt^19^, which showed that 24 genes were significantly enriched within the top 10 disease-associated gene categories (all raw *P*-values < 1 × 10^−2^ and adjusted *P*-values < 1 × 10^−2^), including psychotic disorders, epilepsy, and schizophrenia (Supplementary Information “*CHRM3* Co-Expression Analysis” and Supplementary Table S3). According to GWAS database GRASP^21^, *CHRM3* is an epilepsy risk gene and *CHRM3* SNP rs10925980 was nominally associated with genetic generalized epilepsies (*P* = 1.90 × 10^−6^) and Ca-HL (*P* = 0.03) in EAs (see Supplementary Table S4). In addition, 24 *CHRM3* co-expressed genes were significantly (all *P*-values < 1 × 10^−2^) correlated with *CHRM3* in at least 1 of 10 brain tissues, with the same being true for 14 *Chrm3* co-expressed genes expressed in GABAergic neurons (Supplementary Fig. 4).

The most frequently observed co-expression was between *CHRM3* and *GABRG2*, which was seen in eight brain tissues (all *P*-values < 1 × 10^−10^ and *r*^2^ ≥ 0.6). *CHRM3* was also significantly co-expressed with *CHRNA4* and *HRH3* in THAL (both *P*-values < 1 × 10^−10^ and *r*^2^ ≥ 0.7), and with *HRH3* (*P*-value < 1 × 10^−10^ and *r*^2^ = 0.6) in PUTM. Furthermore, at the single-cell level, the homologous mouse genes *Chrm3* and *Gabrg2* were significantly co-expressed with one another in six different GABAergic neuronal cells (all *P*-values < 1 × 10^−5^ and *r*^2^ ≥ 0.6), whereas *Hrh3* and *Chrna4* were both significantly co-expressed with *Chrm3* at least in three different GABAergic single neurons (all *P*-values < 1 × 10^−5^ and *r*^2^ ≥ 0.4). Taken together, these results show that *CHRM3* is strongly co-expressed with *GABRG2*, *HRH3*, and *CHRNA4* (risk genes for psychotic disorders, epilepsy, and schizophrenia) in THAL and other brain tissues.

## Discussion

We report here biologically interesting GWS results from a case–control GWAS of Ca-HL. There was one GWS association signal at the *CHRM3* locus in Yale-Penn 1 EAs, represented by rs115455482, rs74722579, and rs1938228 (all *r*^2^ ≥ 0.95 in EAs; rs115455482 is rare in AAs), with all three SNPs nominally significant in Yale-Penn 2 EAs. Only rs115455482 and rs74722579 were available in COGA, which when meta-analyzed with Yale-Penn 1 and 2 samples increased the significance of the association. We further evaluated the association of rs115455482 and rs74722579 with Ca-HL in the Yale-Penn 1 and 2, and COGA AA samples. The nominally significant association for rs74722579 (a common variant in both AAs and EAs) in the AA meta-analysis further supports the association. Cis-eQTL analysis of *CHRM3* in brain showed the rs115455482*T risk allele to be associated with lower expression of *CHRM3* in brain tissue THAL. Further co-expression analysis in large brain tissue expression datasets and mouse single neurons demonstrated a significant correlation between the expression of *CHRM3* or its mouse homologous *Chrm3*, and that of three genes (*GABRG2*, *CHRNA4*, and *HRH3*) that have been shown to affect risk for psychotic disorders, including schizophrenia. Similar to *CHRM3* itself, these are all biologically relevant.

The protein product of *CHRM3* is the muscarinic AChR M3, which is localized to multiple tissues, including the brain, smooth muscle, endocrine and exocrine glands, and lungs. In human, mutation of *CHRM3* causes disease of the urinary bladder and a prune-belly-like syndrome^22^. In mice, cannabinoids consistently increase acetylcholine (ACh) and decrease ACh turnover in the HIPP^23^. Pharmacological evidence has also implicated cholinergic dysfunction in the manifestation of psychotic symptoms. Muscarinic ACh receptors (AChRs) play important roles in animal models that are used to examine sensory gating, which is known to be disrupted in schizophrenic patients, and the activation of muscarinic AChRs was suggested as an alternative to classical antipsychotics for the treating of psychotic symptoms^23^. *Chrm3* knockout mice treated with the antipsychotic drug oxotremorine that acts as a selective muscarinic ACh receptor agonist^24–26^ displayed increased dopamine release, which is consistent with *Chrm3* playing an inhibitory role in dopamine release^24^. In animal models, oxotremorine can reverse methamphetamine-, ketamine-, and cocaine-induced hyperlocomotion^26^. Blockade of cholinergic receptors, particularly muscarinic receptors, causes a psychosis characterized by hallucinations and cognitive impairment in normal human subjects and exacerbates symptoms in schizophrenic patients^26^. This is consistent with the assumption that cannabis use is an environmental risk factor in the etiology of schizophrenia, as THC may directly affect T-type calcium channels in the THAL by increasing the excitability of THAL neurons^27^. In our study, the risk rs115455482*T allele is most significantly associated with lower expression of *CHRM3* in THAL, suggesting potential excitatory-to-inhibitory imbalance in the THAL may predispose to cannabis-induced psychosis or schizophrenia.

In the analysis of several brain tissue samples, *CHRM3* expression was associated with the expression of three other genes related to neuropsychiatric traits, *GABRG2*, *CHRNA4*, and *HRH3*. *GABRG2* was co-expressed with *CHRM3* across eight brain tissues and the same co-expression pattern was observed for its mouse homologous *Chrm3* with *Gabrg2* in six GABAergic single neurons. *GABRG2* encodes a γ-aminobutyric acid receptor subunit; the receptor has chloride channel activity. Mutations in *GABRG2* have been associated with epilepsy and febrile seizures^28^. Meanwhile, *CHRNA4* and *HRH3* are co-expressed with *CHRM3* in specific brain tissues (particularly in THAL) and the same pattern is observed for the mouse homologs *Chrna4* and *Hrh3* with different GABAergic neurons. *CHRNA4* encodes CHRNA4 (the α4 nicotinic ACh receptor), which belongs to a superfamily of ligand-gated ion channels that play a role in fast synaptic signal transmission. CHRNA4 interacts with CHRNB2 (the β_2_ nicotinic ACh receptor), which are critical for dopamine-dependent locomotor activation after repeated nicotine administration^29^. Mutations in *CHRNA4* cause nocturnal frontal lobe epilepsy type 1^30^ and polymorphisms of *CHRNA4* have been reported in association with nicotine dependence^31–33^. *HRH3* encodes the histamine receptor H3, which is ubiquitously released from neurons, mast cells, and enterochromaffin-like cells, and can regulate neurotransmitter release^34^. The significant co-expression between *CHRM3* and *GABRG2*, *CHRNA4*, and *HRH3* reflects the critical function of *CHRM3* in the brain, which raises the question of whether, in response to long-term cannabis use, there is crosstalk between *CHRM3* and the three noted genes in Ca-HL.

Multiple lines of evidence support the involvement of *CHRM3* in schizophrenia, which has some symptoms phenotypically close to Ca-HL. In a neural connectivity GWAS in a Chinese schizophrenia case–control sample, *CHRM3* variant rs6700381 was significantly associated with abnormal thalamo-orbital FCTX functional connectivity in first-episode schizophrenia patients^35^. In a multi-stage schizophrenia GWAS of up to 36,989 cases and 113,075 controls^36^, 1 SNP rs72769124 near *CHRM3* was possibly associated with schizophrenia (*P* = 5.5 × 10^−7^; Supplementary Fig. S5). According to the Brainiac database, neither rs6700381 nor rs72769124 is a *CHRM3* eQTL among ten healthy brain tissues. These risk variants may, however, be *CHRM3* eQTLs under different conditions or environmental exposures or in different brain regions, or in different populations.

In our study, despite the lack of independent replication of rs115455482 in COGA, when we meta-analyzed Yale-Penn and COGA samples, the association of rs115455482 genotype with Ca-HL improved. The lack of significant replication in COGA may be due to the differential distributions of cannabis-dependence (CAD) criterion counts in Ca-HL cases and controls between Yale-Penn and COGA samples (Supplementary Fig. S6). We found that Ca-HL was significantly associated with CAD criterion counts both in Yale-Penn and COGA samples (Supplementary Fig. S7), which raises the question of potential involvement of the interaction between rs115455482 genotype and CAD severity in Ca-HL. As shown in Supplementary Fig. S7, we observed that the interaction between rs115455482*T and CAD criterion count was significantly associated with Ca-HL in Yale-Penn 1 EAs (*P* = 1.25 × 10^−5^) but not in Yale-Penn 2 EAs (*P* = 8.94 × 10^−2^) or COGA EAs (*P* = 0.72). This may be a potential reason for the lack of replication of rs115455482 in COGA, as the interaction between rs115455482*T and CAD criterion counts were different among Yale-Penn and COGA samples.

We note also a recent cannabis GWAS publication^37^ implicated *CHRNA2* (and no other locus) as associated with cannabis-use disorder. In our study, *CHRM3* was demonstrated to be significantly co-expressed with *CHRNA4* in the brain tissue THAL. *CHRNA2* and *CHRNA4* encode the nicotinic AChR subunit α-2 and α-4, respectively. Our result considered in that context provides additional weight to considering cholinergic signaling as a mechanism of risk for cannabis-use disorder and cannabis-induced harmful effects.

This study has limitations. The sample size (number of Ca-HL cases) is small in the discovery GWAS, indicating that the power of our GWAS is likely to be low except for risk loci of large effect. Genes close to other prominent SNP association signals (*P* < 5 × 10^−7^) in the meta-analysis of Yale-Penn samples may warrant further study, including *APBA2*, *EFCAB3*, *MTRR*, *PRPRN2*, *SPATA6*, and *FLJ12825* (SNPs mapped to these genes are included in Supplementary Table S5). A continuous measure (e.g., frequency of having experienced hearing, seeing or smelling things that were not really there after marijuana use) would provide more statistical power and should be considered for future surveys eliciting information on the trait.

In summary, we report one GWS association signal, represented by rs115455482 and rs74722579 (*r*^2^ ≥ 0.95 in EAs; rs74722579 is a common variant in both EAs and AAs) at the *CHRM3* locus, with Ca-HL. The findings were GWS in our Yale-Penn 1 taken separately, with nominally significant associations of two variants in one of two other available samples of EAs. Additional support was obtained for rs74722579 in the meta-analysis of AA samples. Our findings are consistent with a model in which the regulatory variant rs115455482 affects the expression of *CHRM3* in THAL, which could alter the stimulatory efficiency of cannabis on hallucinations, a potential underlying mechanism for its association with Ca-HL. Validation of these findings and the putative mechanism is warranted to increase our understanding of the biology of Ca-HL.

## Supplementary information

Supplemental materials
