# Supplementary material for: A regulatory variant of CHRM3 is associated with cannabis-induced hallucinations in European Americans
Source: Transl Psychiatry. 2019 Nov 18;9:309. doi: 10.1038/s41398-019-0639-7 (PMC6861240; doi:10.1038/s41398-019-0639-7)
Supplement: Supplementary file 1 — Supplemental materials [file 41398_2019_639_MOESM1_ESM.docx]

**A Regulatory Variant of *CHRM3* is Associated with Cannabis-Induced Hallucinations in European Americans**

***Supplementary Information***

**Contents**

**Supplementary Figures S1-S7**

**Supplementary Tables S1-S5**

***CHRM3* Co-Expression Analysis**

**The Association of *CHRM3* Variants with Other Diseases**

**The Collaborative Study of the Genetics of Alcoholism (COGA)**

**Supplementary References**

**Supplementary Figures**

**Supplementary Figure S1**

**
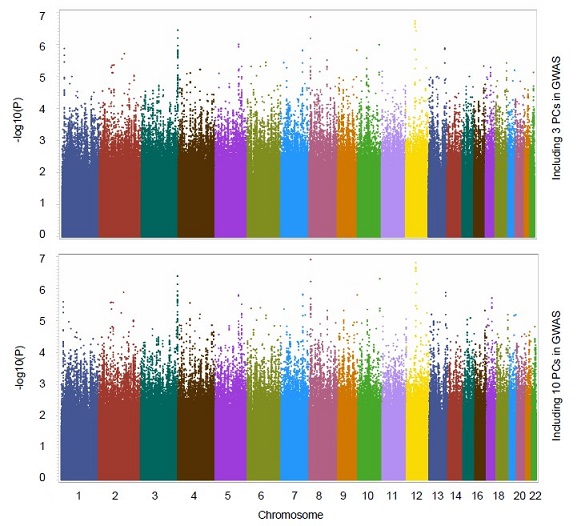
**

**Supplementary Figure S1. Manhattan plots for meta-analysis of cannabis-induced hallucination GWAS in Yale-Penn 1 and 2 African Americans by including 3 PCs as well as other covariates, including sex, age, BMI and sample relatedness.**

**Supplementary Figure S2**


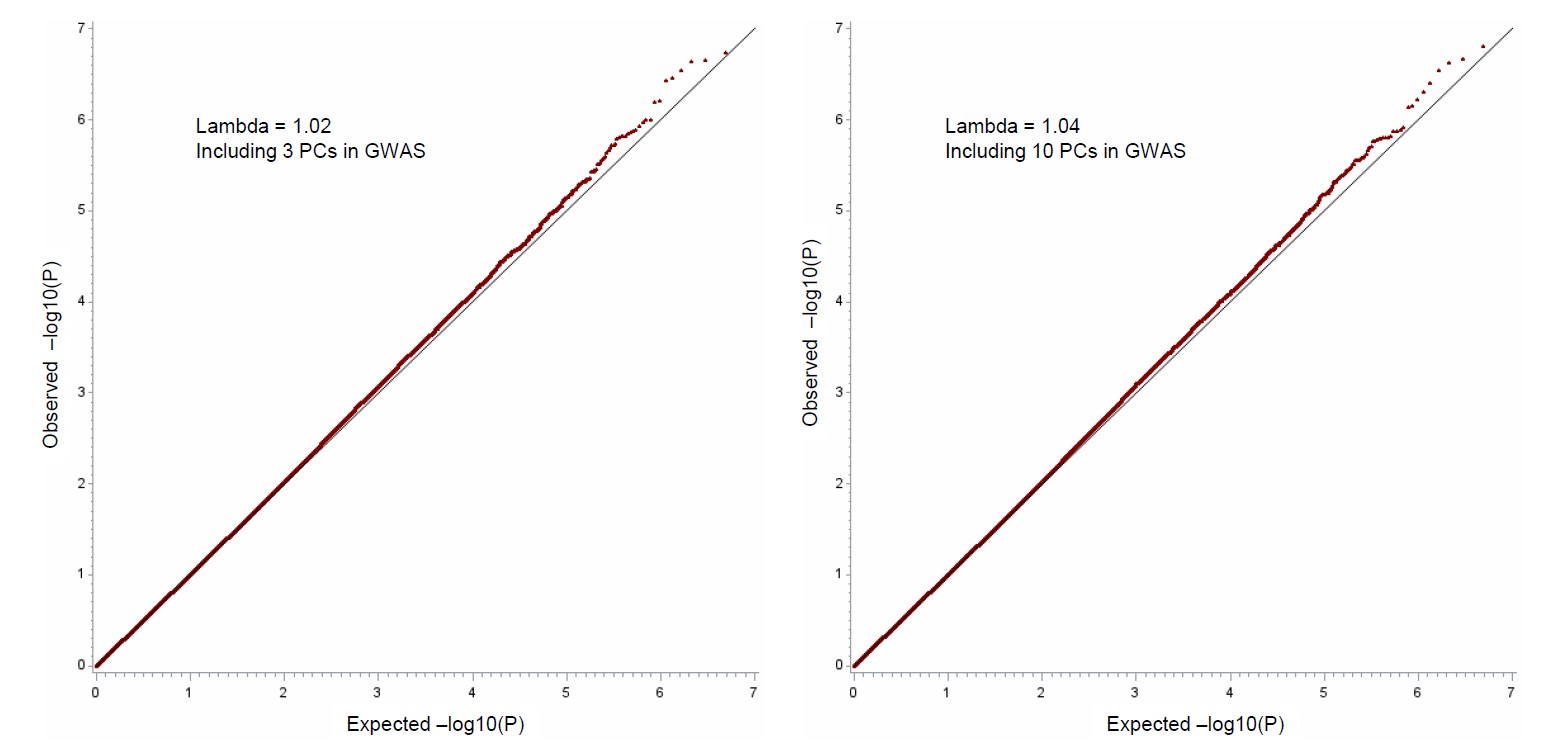


**Supplementary Figure S2. QQ plot for meta-analysis of cannabis-induced hallucination GWAS in Yale-Penn 1 and 2 African Americans by including 3 PCs, as well as other covariates, including sex, age, BMI and sample relatedness.**

**Supplementary Figure S3**

**
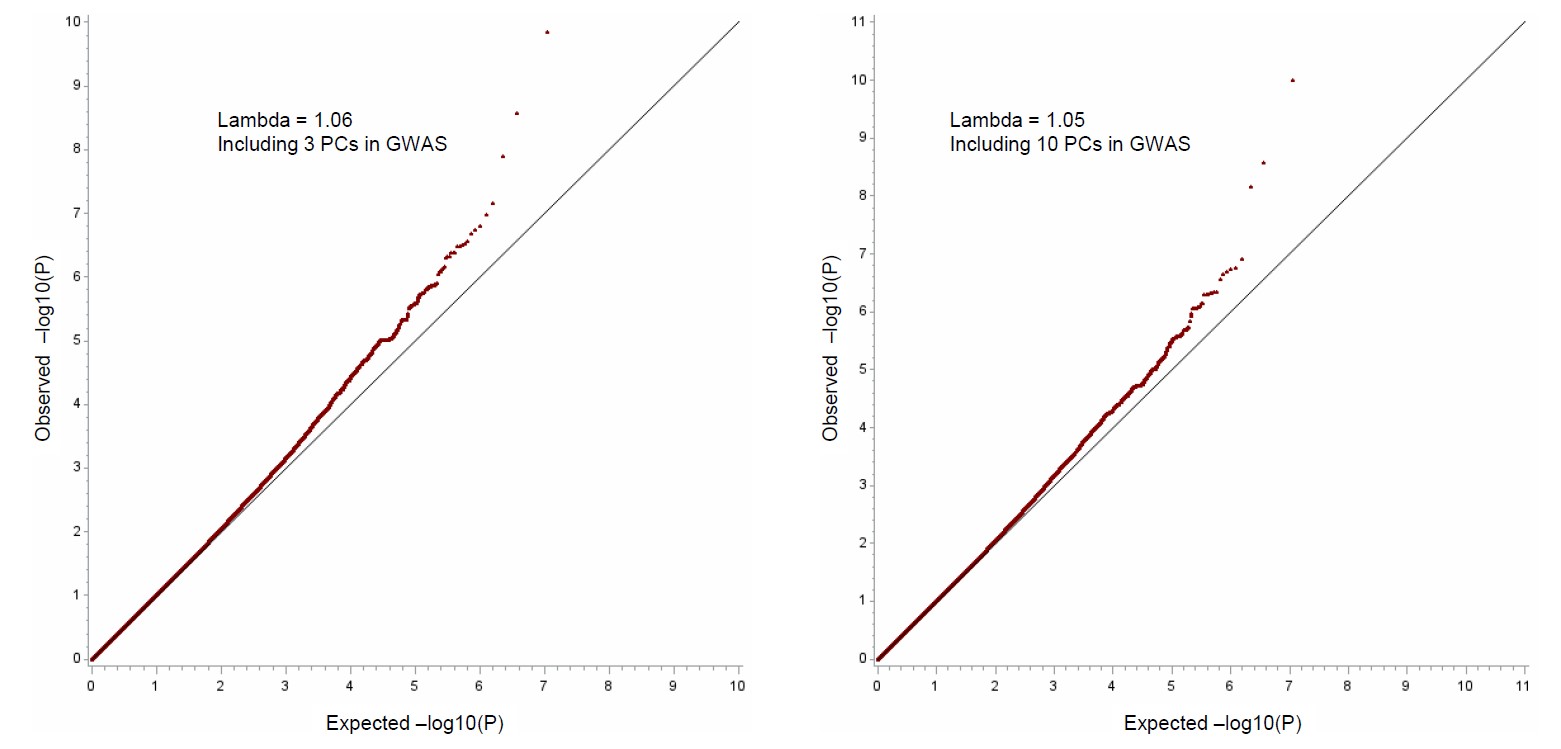
**

**Supplementary Figure S3 QQ plot for meta-analysis of cannabis-induced hallucination GWAS in Yale-Penn 1 and 2 European Americans by including 3 PCs, as well as other covariates, including sex, age, BMI and sample relatedness.**

**Supplementary Figure S4**

**
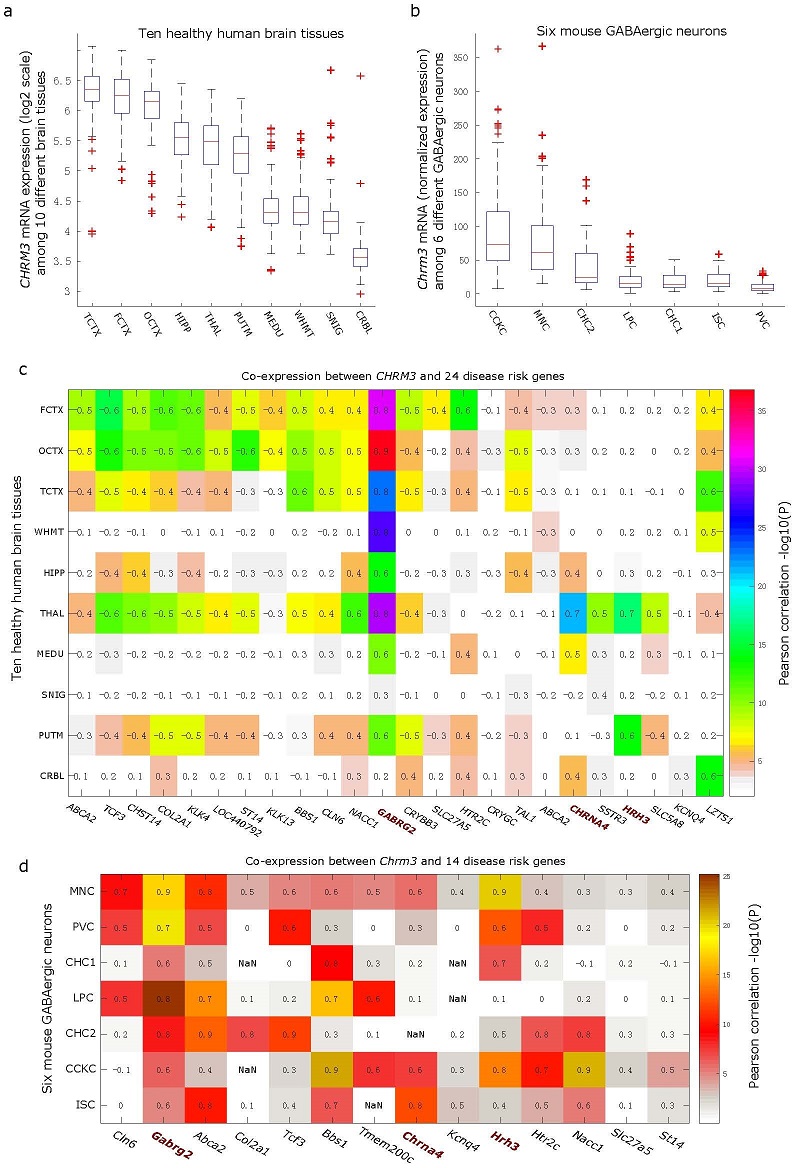
**

**Supplementary Figure S4. The mRNA expression of *CHRM3* and its mouse homologous *Chrm3* were associated with 24 genes across 10 human brain tissues and with 14 mouse genes among 6 GABAergic neurons, respectively.**

*CHRM3* and the homologous mouse gene *Chrm3* are variably expressed among 10 human brain tissues [1] (**a**) and 6 types of GABAergic neurons [2] (**b**), respectively. Pairwise Pearson correlation coefficients (r) for *CHRM3* and 24 genes are depicted in the heatmap (**c**), while, the same correlation analysis between mouse gene *Chrm3* and 14 mouse homologous genes out of these 24 human genes across 6 types of GABAergic neurons are displayed in (**d**). The -log10(*P*) correlation *P* values are indicated by different colors, with its corresponding Pearson correlation coefficient labeled in each square of the heatmaps; the NaN represents the gene is not expressed in specific cell type and cannot be correlated with *Chrm3*.

Note: line within each boxplot represents median, and box region indicates the range from first quantile to third quantile. Up and down whiskers, as well as pluses outside box region, represent maximum value, minimum value, and outliers, respectively. The abbreviations for 10 brain tissues are CRBL (cerebellar cortex), FCTX (frontal cortex), HIPP (hippocampus), MEDU (medulla specifically inferior olivary nucleus), OCTX (occipital cortex), PUTM (putamen), SNIG (substantia nigra), TCTX (temporal cortex), THAL (thalamus) and WHMT (intralobular white matter). The six types of neurons are martinotti cells (MNC), interneuron selective cells (ISC), CCK-basket cells (CCKC), PV basket cells (PVC), chandelier cells upper layer (CHC1) and deep layer (CHC2) of mouse brain, and long projecting cells (LPC), all of which were isolated from mouse frontal cortex.

**Supplemental Figure S5**


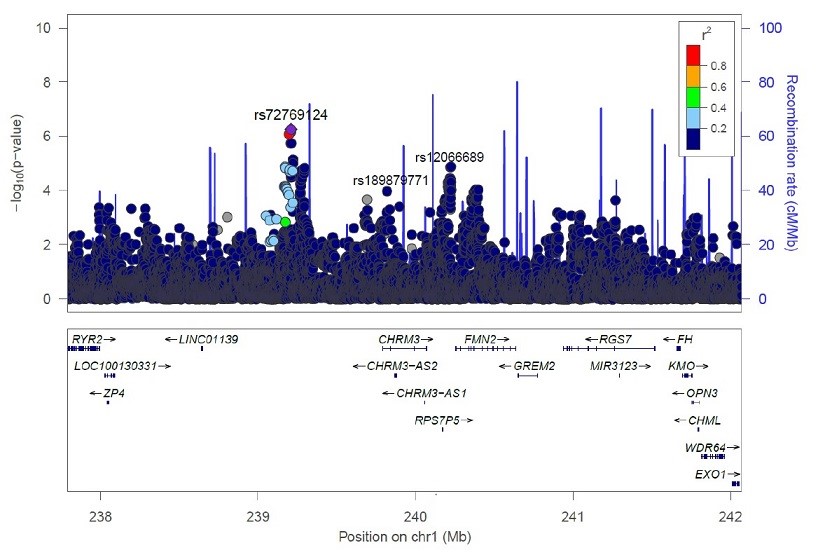


**Supplementary Figure S5. Local Manhattan plot showing SNPs near gene *CHRM3* and their association with schizophrenia.** According to a published schizophrenia genome-wide association study [3], three SNPs, including rs72769124, rs12066689 and rs189879771, were not highly correlated with each other (all r^2^ <0.2 in EAs) and were nominally associated with schizophrenia (*P* values 5.5×10^-7^, 1.3×10^-6^, and 1.1×10^-4^, respectively). Rs72769124 and rs12066689 were not associated with cannabis-induced hallucinations (Ca-HL) in European Americans (EAs) (all *P* values >0.5). Other SNPs near the association peak of rs72769124 were nominally associated with schizophrenia (all *P* values<1×10^-5^) but not associated with Ca-HL (all *P* values >0.87). Meanwhile, rs189879771, residing in the intronic region of *CHRM3*, was also not significantly associated with Ca-HL in EAs (*P*= 0.09). The light blue line and right Y-axis show the observed recombination rate in the 1000 Genomes Project European samples (EUR, hg19). The SNPs are colored in accordance to r^2^ with rs72769124.

**Supplementary Figure S6**


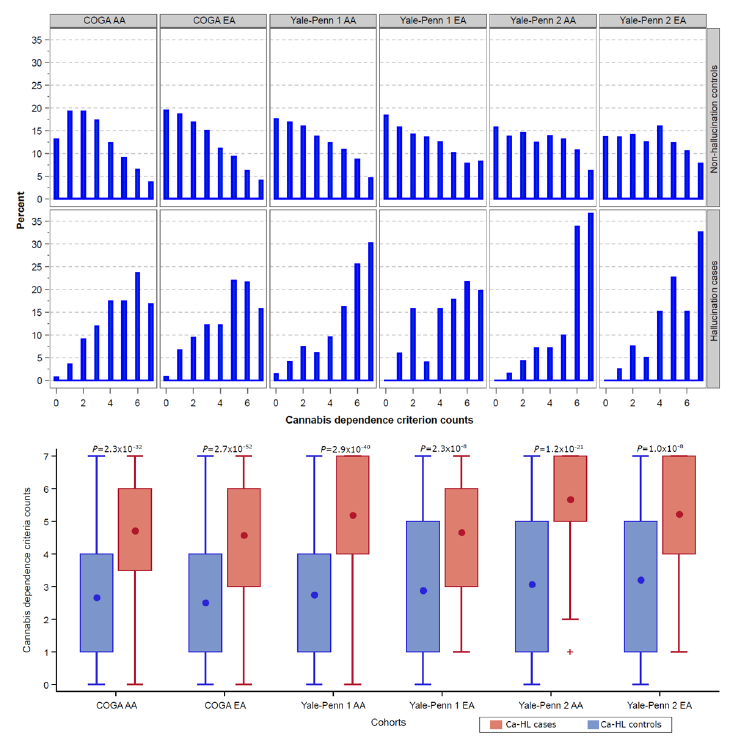


**Supplementary Figure S6. Cannabis dependence (CAD) criterion counts were differentially distributed between cases and controls of cannabis-induced hallucinations.**

In the upper panel, the percentages of CAD criterion counts ranging from 0 to 7 for cannabis-induced hallucination (Ca-HL) cases and controls across different subsamples are summarized in histograms. Meanwhile, in the lower panel, the significant difference of CAD criterion counts in Ca-HL cases and controls is demonstrated for each cohort. Dot within each boxplot represents mean, and box region indicates the range from first quantile to third quantile. Up and down whiskers represent maximum value and minimum value, respectively.

**Supplementary Figure S7**


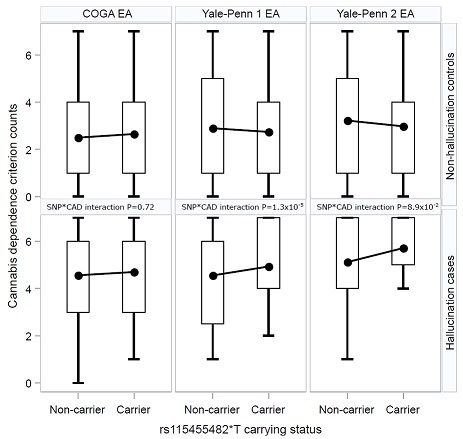


**Supplementary Figure S7. The interaction between rs115455482*T allele and cannabis dependence criterion counts affects cannabis-induced hallucinations.** Boxplots illustrate the interaction between rs115455482*T allele and cannabis dependence (CAD) criterion counts in Yale-Penn and COGA European Americans (EAs). To determine the interaction between *CHRM3* SNP rs115455482 and CAD criterion counts, GEMMA [4] command ‘-gxe’ was used to fit a linear mixed model to control both the SNP main effect and the effect of CAD criterion counts, adjust sex, age, the first three PCs of ancestry, and the degree of relatedness among subjects, and test the interaction effect between rs115455482*T and CAD criterion counts. The association of the interaction between rs115455482*T and CAD criterion counts with Ca-HL was significant in Yale-Penn 1 EAs (*P*=1.3×10^-5^), marginally significant in Yale-Penn 2 EAs (*P*=8.9×10^-2^), and not significant in COGA EAs (*P*=0.72).

Dot within each boxplot represents mean, and box region indicates the range from first quantile to third quantile. Up and down whiskers represent maximum value and minimum value, respectively.

**Supplementary Table S1. Linkage disequilibrium (LD) for rs115455482 and rs74722579 among European and African populations.**

| **Query SNP** | **chr** | **pos (hg38)** | **LD** | **LD** | **variant** | **Ref** | **Alt** | **AFR** | **AMR** | **ASN** | **EUR** | **Motifs** |
| --- | --- | --- | --- | --- | --- | --- | --- | --- | --- | --- | --- | --- |
|  |  |  | **(r²)** | **(D')** |  |  |  | **freq** | **freq** | **freq** | **freq** | **changed** |
| rs115455482 (r2>=0.8) in EUR | 1 | 239386955 | 0.93 | 1 | [rs117549427](http://archive.broadinstitute.org/mammals/haploreg/detail_v4.1.php?query=&id=rs117549427) | G | A | 0.01 | 0.01 | 0.05 | 0.06 | 9 altered motifs |
|  | 1 | 239393278 | 0.95 | 1 | [rs60093138](http://archive.broadinstitute.org/mammals/haploreg/detail_v4.1.php?query=&id=rs60093138) | A | G | 0.27 | 0.07 | 0.15 | 0.06 | 6 altered motifs |
|  | 1 | 239397249 | 0.95 | -1 | [rs1938228](http://archive.broadinstitute.org/mammals/haploreg/detail_v4.1.php?query=&id=rs1938228) | T | C | 0.70 | 0.96 | 0.95 | 0.94 |  |
|  | 1 | 239399460 | 1 | 1 | [rs115455482](http://archive.broadinstitute.org/mammals/haploreg/detail_v4.1.php?query=&id=rs115455482) | C | T | 0.01 | 0.02 | 0.05 | 0.06 | 4 altered motifs |
|  | 1 | 239401207 | 0.95 | 1 | [rs74722579](http://archive.broadinstitute.org/mammals/haploreg/detail_v4.1.php?query=&id=rs74722579) | G | C | 0.10 | 0.02 | 0.05 | 0.06 | Irf,ZEB1 |
|  |  |  |  |  |  |  |  |  |  |  |  |  |
| rs74722579 (r2>=0.2) in AFR | 1 | 239393278 | 0.29 | 1 | [rs60093138](http://archive.broadinstitute.org/mammals/haploreg/detail_v4.1.php?query=&id=rs60093138) | A | G | 0.27 | 0.07 | 0.15 | 0.06 | 6 altered motifs |
|  | 1 | 239396048 | 0.24 | 1 | [rs4130463](http://archive.broadinstitute.org/mammals/haploreg/detail_v4.1.php?query=&id=rs4130463) | C | T | 0.32 | 0.11 | 0.38 | 0.09 | 6 altered motifs |
|  | 1 | 239396169 | 0.24 | 1 | [rs7552265](http://archive.broadinstitute.org/mammals/haploreg/detail_v4.1.php?query=&id=rs7552265) | A | G | 0.32 | 0.11 | 0.38 | 0.09 | PPAR,RXRA |
|  | 1 | 239397249 | 0.3 | -1 | [rs1938228](http://archive.broadinstitute.org/mammals/haploreg/detail_v4.1.php?query=&id=rs1938228) | T | C | 0.70 | 0.96 | 0.95 | 0.94 |  |
|  | 1 | 239400093 | 0.32 | 1 | [rs114292606](http://archive.broadinstitute.org/mammals/haploreg/detail_v4.1.php?query=&id=rs114292606) | T | A | 0.03 | 0 | 0 | 0 | 4 altered motifs |
|  | 1 | 239401207 | 1 | 1 | [rs74722579](http://archive.broadinstitute.org/mammals/haploreg/detail_v4.1.php?query=&id=rs74722579) | G | C | 0.10 | 0.02 | 0.05 | 0.06 | Irf,ZEB1 |

**Note:** By querying rs115455482 and rs74722579 in the Haploreg4 database [5], it was demonstrated that these two SNPs are two common variants highly linked (r^2^>0.9) in European but not African populations. Rs74722579 but not rs115455482 is a common variant in African population.

**Supplementary Table S2. Top 100 co-expressed genes of *CHRM3* identified by COXPRESdb [6] and ranked by its correlation value with *CHRM3*.**

| **Gene** | **Function** | **Chr*** | **Entrez Gene ID** |
| --- | --- | --- | --- |
| *CHRM3* | cholinergic receptor, muscarinic 3 | Chr1 | 1131 |
| *SLC27A5* | solute carrier family 27 (fatty acid transporter), member 5 | Chr19 | 10998 |
| *SPATA3* | spermatogenesis associated 3 | Chr2 | 130560 |
| *CBX2* | chromobox homolog 2 | Chr17 | 84733 |
| *MAPT-AS1* | MAPT antisense RNA 1 | Chr17 | 100128977 |
| *LOC255654* | uncharacterized LOC255654 | Chr1 | 255654 |
| *HTR2C* | 5-hydroxytryptamine (serotonin) receptor 2C, G protein-coupled | ChrX | 3358 |
| *NUTM2F* | NUT family member 2F | Chr9 | 54754 |
| *OXGR1* | oxoglutarate (alpha-ketoglutarate) receptor 1 | Chr13 | 27199 |
| *SHC4* | SHC (Src homology 2 domain containing) family, member 4 | Chr15 | 399694 |
| *LZTS1* | leucine zipper, putative tumor suppressor 1 | Chr8 | 11178 |
| *MGAT3* | mannosyl (beta-1,4-)-glycoprotein beta-1,4-N-acetylglucosaminyltransferase | Chr22 | 4248 |
| *LINC01139* | long intergenic non-protein coding RNA 1139 | Chr1 | 339535 |
| *BBS1* | Bardet-Biedl syndrome 1 | Chr11 | 582 |
| *RNF207* | ring finger protein 207 | Chr1 | 388591 |
| *MYO7B* | myosin VIIB | Chr2 | 4648 |
| *PPP1R12C* | protein phosphatase 1, regulatory subunit 12C | Chr19 | 54776 |
| *ZSCAN5A* | zinc finger and SCAN domain containing 5A | Chr19 | 79149 |
| *ZNF707* | zinc finger protein 707 | Chr8 | 286075 |
| *KLK4* | kallikrein-related peptidase 4 | Chr19 | 9622 |
| *ARHGAP33* | Rho GTPase activating protein 33 | Chr19 | 115703 |
| *ST14* | suppression of tumorigenicity 14 (colon carcinoma) | Chr11 | 6768 |
| *KIAA1683* | KIAA1683 | Chr19 | 80726 |
| *LOC100131347* | RAD52 motif containing 1 pseudogene | Chr17 | 100131347 |
| *KLHDC4* | kelch domain containing 4 | Chr16 | 54758 |
| *LTBP3* | latent transforming growth factor beta binding protein 3 | Chr11 | 4054 |
| *SUSD3* | sushi domain containing 3 | Chr9 | 203328 |
| *BCL6B* | B-cell CLL/lymphoma 6, member B | Chr17 | 255877 |
| *HRASLS2* | HRAS-like suppressor 2 | Chr11 | 54979 |
| *MAPK8IP3* | mitogen-activated protein kinase 8 interacting protein 3 | Chr16 | 23162 |
| *ZDHHC1* | zinc finger, DHHC-type containing 1 | Chr16 | 29800 |
| *ABCA2* | ATP-binding cassette, sub-family A (ABC1), member 2 | Chr9 | 20 |
| *TRAF7* | TNF receptor-associated factor 7, E3 ubiquitin protein ligase | Chr16 | 84231 |
| *KLK13* | kallikrein-related peptidase 13 | Chr19 | 26085 |
| *ODAM* | odontogenic, ameloblast asssociated | Chr4 | 54959 |
| *KREMEN1* | kringle containing transmembrane protein 1 | Chr22 | 83999 |
| *PRR4* | proline rich 4 (lacrimal) | Chr12 | 11272 |
| *CYTH3* | cytohesin 3 | Chr7 | 9265 |
| *ADAD2* | adenosine deaminase domain containing 2 | Chr16 | 161931 |
| *KLHDC10* | kelch domain containing 10 | Chr7 | 23008 |
| *ART3* | ADP-ribosyltransferase 3 | Chr4 | 419 |
| *PDE4A* | phosphodiesterase 4A, cAMP-specific | Chr19 | 5141 |
| *GABRG2* | gamma-aminobutyric acid (GABA) A receptor, gamma 2 | Chr5 | 2566 |
| *DKFZP434A062* | uncharacterized LOC26102 | Chr9 | 26102 |
| *OBSL1* | obscurin-like 1 | Chr2 | 23363 |
| *EMX1* | empty spiracles homeobox 1 | Chr2 | 2016 |
| *SNTB2* | syntrophin, beta 2 (dystrophin-associated protein A1, 59kDa, basic component 2) | Chr16 | 6645 |
| *SSTR3* | somatostatin receptor 3 | Chr22 | 6753 |
| *NPAS4* | neuronal PAS domain protein 4 | Chr11 | 266743 |
| *ADAMTSL5* | ADAMTS-like 5 | Chr19 | 339366 |
| *BOK* | BCL2-related ovarian killer | Chr2 | 666 |
| *LOC399884* | uncharacterized LOC399884 | Chr11 | 399884 |
| *TSSK6* | testis-specific serine kinase 6 | Chr19 | 83983 |
| *RGS6* | regulator of G-protein signaling 6 | Chr14 | 9628 |
| *ROPN1* | rhophilin associated tail protein 1 | Chr3 | 54763 |
| *TMEM132A* | transmembrane protein 132A | Chr11 | 54972 |
| *OSBPL10* | oxysterol binding protein-like 10 | Chr3 | 114884 |
| *TMEM200C* | transmembrane protein 200C | Chr18 | 645369 |
| *SOX8* | SRY (sex determining region Y)-box 8 | Chr16 | 30812 |
| *RNF151* | ring finger protein 151 | Chr16 | 146310 |
| *ADRA2B* | adrenoceptor alpha 2B | Chr2 | 151 |
| *LTBR* | lymphotoxin beta receptor (TNFR superfamily, member 3) | Chr12 | 4055 |
| *ADAMTS7* | ADAM metallopeptidase with thrombospondin type 1 motif, 7 | Chr15 | 11173 |
| *CRYBB3* | crystallin, beta B3 | Chr22 | 1417 |
| *SOSTDC1* | sclerostin domain containing 1 | Chr7 | 25928 |
| *CHRNA4* | cholinergic receptor, nicotinic, alpha 4 (neuronal) | Chr20 | 1137 |
| *NEU2* | sialidase 2 (cytosolic sialidase) | Chr2 | 4759 |
| *ENDOV* | endonuclease V | Chr17 | 284131 |
| *LOC100131283* | uncharacterized LOC100131283 | Chr6 | 100131283 |
| *KCNQ4* | potassium voltage-gated channel, KQT-like subfamily, member 4 | Chr1 | 9132 |
| *ZNF696* | zinc finger protein 696 | Chr8 | 79943 |
| *ADCY1* | adenylate cyclase 1 (brain) | Chr7 | 107 |
| *TCF3* | transcription factor 3 | Chr19 | 6929 |
| *EXOG* | endo/exonuclease (5'-3'), endonuclease G-like | Chr3 | 9941 |
| *H1FNT* | H1 histone family, member N, testis-specific | Chr12 | 341567 |
| *NACC1* | nucleus accumbens associated 1, BEN and BTB (POZ) domain containing | Chr19 | 112939 |
| *SLC5A8* | solute carrier family 5 (sodium/monocarboxylate cotransporter), member 8 | Chr12 | 160728 |
| *LOC339539* | uncharacterized LOC339539 | Chr1 | 339539 |
| *TAL1* | T-cell acute lymphocytic leukemia 1 | Chr1 | 6886 |
| *CBLC* | Cbl proto-oncogene C, E3 ubiquitin protein ligase | Chr19 | 23624 |
| *NANOS3* | nanos homolog 3 (Drosophila) | Chr19 | 342977 |
| *CRYGC* | crystallin, gamma C | Chr2 | 1420 |
| *WNT8B* | wingless-type MMTV integration site family, member 8B | Chr10 | 7479 |
| *ZNF777* | zinc finger protein 777 | Chr7 | 27153 |
| *LINGO2* | leucine rich repeat and Ig domain containing 2 | Chr9 | 158038 |
| *CLN6* | ceroid-lipofuscinosis, neuronal 6, late infantile, variant | Chr15 | 54982 |
| *DNM1P33* | DNM1 pseudogene 33 | Chr15 | 554175 |
| *FOXK1* | forkhead box K1 | Chr7 | 221937 |
| *SIK1* | salt-inducible kinase 1 | Chr21 | 150094 |
| *HRH3* | histamine receptor H3 | Chr20 | 11255 |
| *LOC440792* | proline dehydrogenase (oxidase) 1 pseudogene | Chr22 | 440792 |
| *HGC6.3* | uncharacterized LOC100128124 | Chr6 | 100128124 |
| *TTC23* | tetratricopeptide repeat domain 23 | Chr15 | 64927 |
| *FLJ31713* | uncharacterized protein FLJ31713 | Chr9 | 158263 |
| *TRIM2* | tripartite motif containing 2 | Chr4 | 23321 |
| *RBM17* | RNA binding motif protein 17 | Chr10 | 84991 |
| *LOC653602* | uncharacterized LOC653602 | Chr2 | 653602 |
| *FREM2* | FRAS1 related extracellular matrix protein 2 | Chr13 | 341640 |
| *SP8* | Sp8 transcription factor | Chr7 | 221833 |
| *COL2A1* | collagen, type II, alpha 1 | Chr12 | 1280 |

* Chr represents chromosome.

**Supplementary Table S3. Disease enrichment among top 100 co-expressed genes of *CHRM3*.**

| **Disease** | **Reference genes (n)** | **Observed genes (n)** | **Expected genes (n)** | **Ratio of enrichment** | **P^1^** | **AdjP^2^** | **Genes in the disease** |
| --- | --- | --- | --- | --- | --- | --- | --- |
| Psychotic Disorders | 129 | 4 | 0.3 | 13.51 | 2.00E-04 | 3.80E-03 | *CHRM3 GABRG2 TMEM200C HTR2C* |
| Prostatic Neoplasms | 262 | 5 | 0.6 | 8.31 | 4.00E-04 | 3.80E-03 | *KLK4*  *ST14*  *SLC5A8*  *LZTS1*  *SSTR3* |
| Blindness | 172 | 4 | 0.39 | 10.13 | 7.00E-04 | 4.30E-03 | *CRYGC*  *BBS1*  *CLN6*  *COL2A1* |
| Nervous System Diseases | 694 | 7 | 1.59 | 4.39 | 1.10E-03 | 4.30E-03 | *GABRG2 KCNQ4 CHRNA4*  *BBS1*  *HTR2C*  *CLN6*  *ABCA2* |
| Epilepsy | 201 | 4 | 0.46 | 8.67 | 1.20E-03 | 4.30E-03 | *GABRG2 CHRNA4 HTR2C*  *CLN6* |
| Schizophrenia | 360 | 5 | 0.83 | 6.05 | 1.50E-03 | 4.30E-03 | *GABRG2 CHRNA4 HRH3*  *HTR2C LOC440792* |
| Eye Diseases | 368 | 5 | 0.84 | 5.92 | 1.60E-03 | 4.30E-03 | *CHRM3 CRYGC CRYBB3*  *BBS1*  *COL2A1* |
| Translocation, Genetic | 431 | 5 | 0.99 | 5.05 | 3.20E-03 | 6.20E-03 | *SLC27A5 HTR2C SLC5A8*  *TCF3*  *TAL1* |
| Urogenital Neoplasms | 432 | 5 | 0.99 | 5.04 | 3.30E-03 | 6.20E-03 | *KLK4*  *KLK13*  *NACC1*  *ST14*  *LZTS1* |
| Neoplasms | 854 | 7 | 1.96 | 3.57 | 3.60E-03 | 6.20E-03 | *KLK4*  *KLK13*  *NACC1*  *ST14*  *SLC5AB*  *LZTS1*  *TAL1* |

1. Hypergeometric test P value. 2. Hypergeometric test after multiple test adjustment P value.

**Supplementary Table S4. The association signals of *CHRM3* SNPs reported in other GWAS and their corresponding association signals in the cannabis-induced hallucination GWAS in European Americans (EAs).**

| Cannabis-induced hallucination GWAS (EAs) | | | *CHRM3* signals reported in other GWAS | | | |
| --- | --- | --- | --- | --- | --- | --- |
| **dbSNP** | **P** | **Beta** | **Pvalue** | **PMID** | **Phenotype** | **GWAS** |
| rs12059546 | 0.09 | 0.14 | 4.10E-08 | 22949513 | Genetic generalized epilepsis | European |
| rs1110615 | 0.06 | 0.24 | 6.60E-08 | 22949513 | Genetic generalized epilepsis | European |
| rs1591887 | 0.04 | 0.14 | 2.60E-07 | 22949513 | Genetic generalized epilepsis | European |
| rs12063454 | 0.06 | 0.12 | 2.80E-07 | 22949513 | Genetic generalized epilepsis | European |
| rs12077627 | 0.06 | 0.12 | 2.90E-07 | 22949513 | Genetic generalized epilepsis | European |
| rs10925980 | 0.03 | 0.07 | 1.90E-06 | 22949513 | Genetic generalized epilepsis | European |
| rs2355230 | 0.10 | 0.38 | 9.20E-10 | 21694509 | Postoperative nausea and vomiting | European |
| rs2165870 | 0.10 | 0.38 | 5.50E-05 | 21694509 | Postoperative nausea and vomiting | European |
| rs12034521 | 0.76 | 0.76 | 5.10E-06 | 20663923 | Autism | Mixed |
| rs6678395 | 0.47 | 0.87 | 8.60E-05 | 19181680 | Bone mineral content | European |
| rs6671410 | 0.71 | 0.78 | 2.30E-06 | 21826061 | Crystallized intelligence (combination of multiple tests) | European |
| rs2820037 | 0.27 | 0.27 | 7.70E-07 | 17554300 | Hypertension | European |
| rs2820037 | 0.27 | 0.27 | 8.00E-07 | 21347282 | Hypertension | African |
| rs2820038 | 0.27 | 0.27 | 9.10E-07 | 17554300 | Hypertension | European |
| rs2790622 | 0.27 | 0.27 | 9.40E-07 | 17554300 | Hypertension | European |
| rs2820046 | 0.29 | 0.29 | 1.10E-06 | 17554300 | Hypertension | European |
| rs9428826 | 0.29 | 0.29 | 2.30E-06 | 17554300 | Hypertension | European |
| rs2820026 | 0.52 | 0.52 | 3.40E-06 | 17554300 | Hypertension | European |
| rs1578180 | 0.12 | 0.33 | 3.80E-05 | 21130836 | Information processing speed (Digit symbol) | European |
| rs1072319 | 0.12 | 0.33 | 3.80E-05 | 21130836 | Information processing speed (Digit symbol) | European |
| rs946355 | 0.88 | 0.88 | 2.60E-05 | 19902172 | Insulin disposition index | Hispanic |
| rs10754677 | 0.92 | 0.96 | 3.40E-05 | 21658281 | Myocardial infarction (MI), sudden cardiac arrest in patients with coronary artery disease (CAD) | European |
| rs10925917 | 0.33 | 0.81 | 7.30E-05 | 21658281 | Myocardial infarction (MI), sudden cardiac arrest in patients with coronary artery disease (CAD) | European |

Note: GRASP Search - v2.0.0.0 (https://grasp.nhlbi.nih.gov/Search.aspx) was used to search for genome-wide association studies (GWASs) having *CHRM3* variants that passed the association threshold of *P*<1E-04. These variants were then queried in our cannabis-induced hallucination GWAS of European Americans only, as most of these GWAS variants are from European population.

**Supplementary Table S5. Other top nominally significant SNPs in the cannabis-induced hallucination GWAS of European Americans (EAs) and African Americans (AAs).**

| **Genomic position** | **Related Gene** | **Chr** | **P** | **BETA** | **dbSNP** | **GWAS** |
| --- | --- | --- | --- | --- | --- | --- |
| intronic | APBA2 | 15 | 1.77E-07 | 0.0565 | rs1123423 | EA |
| intronic | APBA2 | 15 | 2.10E-07 | 0.0575 | rs80318641 | EA |
| intronic | APBA2 | 15 | 4.08E-07 | 0.0542 | rs4779774 | EA |
| intronic | APBA2 | 15 | 4.16E-07 | 0.0563 | rs77925279 | EA |
| intronic | APBA2 | 15 | 4.78E-07 | 0.051 | rs59831373 | EA |
| intergenic | EFCAB3 | 4 | 2.75E-07 | 0.0794 | rs12953094 | EA |
| intergenic | EFCAB3 | 4 | 3.18E-07 | 0.083 | rs13216364 | EA |
| intergenic | LOC151121(dist=358659),LOC389033(dist=290312) | 2 | 4.66E-07 | 0.0816 | rs62353062 | EA |
| intergenic | MTRR(dist=30046),LOC729506(dist=402315) | 5 | 1.54E-07 | 0.0879 | rs76820885 | EA |
| intergenic | MTRR(dist=31009),LOC729506(dist=401352) | 5 | 4.16E-07 | 0.0861 | rs76433132 | EA |
| intronic | PTPRN2 | 7 | 4.87E-07 | 0.0642 | rs142876798 | EA |
| intronic | SPATA6 | 1 | 7.05E-08 | 0.0923 | rs62353096 | EA |
| intronic | SPATA6 | 1 | 1.03E-07 | 0.0908 | rs62353098 | EA |
| intronic | SPATA6 | 1 | 2.96E-07 | 0.0914 | rs184760481 | EA |
| intronic | SPATA6 | 1 | 3.26E-07 | 0.0854 | rs76005938 | EA |
| ncRNA_intronic | FLJ12825 | 12 | 2.85E-07 | 0.0976 | rs116099720 | AA |
| ncRNA_intronic | FLJ12825 | 12 | 2.20E-07 | 0.1002 | rs114771592 | AA |
| ncRNA_intronic | FLJ12825 | 12 | 2.25E-07 | 0.1014 | rs79417529 | AA |
| intergenic | SLC16A7(dist=1159672),FAM19A2(dist=758722) | 12 | 3.73E-07 | 0.0855 | rs74096290 | AA |
| intergenic | ACAP2(dist=4778),MIR5692C1(dist=40050) | 3 | 3.50E-07 | 0.1038 | rs112142845 | AA |
| intergenic | CSMD1(dist=682919),LOC100287015(dist=725830) | 8 | 1.38E-07 | 0.0675 | rs61686001 | AA |

Note: SNPs with association signals passed the threshold of P<5E-07 are displayed.

***CHRM3* Co-Expression Analysis**

Disease enrichment analysis by using WEB-based WebGestalt (the 2013 version) [7] for the top 100 *CHRM3* co-expressed genes (**Supplementary Table S2**) demonstrated that 24 *CHRM3* co-expressed genes as well as *CHRM3* itself (**Supplementary Table S3**) are significantly enriched within 10 diseases (all raw *P* values<1×10^-2^ and adjusted *P* values<1×10^-2^). These enriched diseases include psychotic disorders, schizophrenia, epilepsy, nervous system diseases, eye diseases, prostatic neoplasms, blindness, genetic translocation and urogenital neoplasms and neoplasms. Four genes, including *CHRM3*, *GABRG2*, *TMEM200C* and *HTR2C*, are enriched for psychotic disorders with the most significant disease enrichment *P* value (raw *P*=2.0×10^-4^ and adjusted *P* value=3.8×10^-3^). Interestingly, another two enriched diseases are epilepsy (*CHRM3* co-expressed genes: *GABRG2*, *CHRNA4*, *HTR2C* and *CLN6*) and schizophrenia (*CHRM3* co-expressed genes: *GABRG2*, *CHRNA4*, *HRH3*, *HTR2C* and *LOC440792*), suggesting the potential role of *CHRM3* in these diseases.

We further correlated the expression of these 24 disease risk genes with that of *CHRM3* or its mouse homologous *Chrm3* across 10 brain tissues described in the brain eQTL analysis (total samples=1,340) [1] or 6 GABAergic neurons isolated from mouse frontal cortex (total samples=584) [2], respectively. The expression data were specifically downloaded from Braineac database and the NCBI gene expression omnibus (GEO; accession No. GSE92522). These 6 GABAergic neurons are martinotti cells (MNC), interneuron selective cells (ISC), CCK-basket cells (CCKC), PV basket cells (PVC), chandelier cells upper layer (CHC1) and deep layer (CHC2) of mouse brain, and long projecting cells (LPC). *CHRM3* or its mouse homologous *Chrm3* was variably expressed either in 10 brain tissues (**Supplementary Figure 4a**) or among 6 mouse GABAergic neurons (**Supplementary Figure 4b**). We validated these co-expressed genes of *CHRM3* or its mouse homologue *Chrm3* at tissue level and single cell level, respectively. In Pearson correlation analysis, all these 24 *CHRM3* co-expressed genes were nominally significantly (all *P* values<1×10^-2^) correlated with *CHRM3* at least in one of 10 brain tissues (**Supplementary Figure 4c**), and the same is true for 14 *Chrm3* co-expressed genes that expresses in GABAergic neurons. Notably, these 14 mouse genes were significantly co-expressed with *Chrm3* in the MNC neuron cells. In CCKC neuron cells, 12 out of these 14 genes were strongly co-expressed with *Chrm3* (**Supplementary Figure 4d**). The most significant correlation was observed between *CHRM3* and the *GABRG2* among 8 brain tissues (all *P* values<1×10^-10^ and r^2^>= 0.6), while the correlation was still significant but with lower correlation signals in other 2 brain tissues, including SNIG (*P*<1×10^-5^ and r^2^=0.3) and CRBL (*P*<1×10^-2^ and r^2^=0.2), where *CHRM3* expression were relatively lower than that of other 8 brain tissues (**Supplementary Figure 4a**). For mouse homologous *Chrm3* and *Gabrg2*, the same ubiquitous co-expression was also observed at cellular level among 6 GABAergic neurons, including CCKC, MNC, PVC, CHC, LPC and ISC (all *P* values<1×10^-5^ and r^2^>0.6; only 14 mouse homologous genes out of these 24 co-expressed gene of *CHRM3* were expressed at least in 3 out of 6 GABAergic neurons; **Supplementary Figure 4d**). This widespread co-expression between *CHRM3* and *GABRG2* is noteworthy considering in the context of the fact that *GABRG2* is a risk gene for multiple diseases, including psychotic disorders, schizophrenia, epilepsy, and nervous system diseases (**Supplementary Table S3**). We also observed that in THAL *CHRM3* was highly co-expressed with *CHRNA4* and *HRH3* (both *P* values<1×10^-10^ and r^2^=0.7), both of which are schizophrenia risk genes, further supporting the potential involvement of *CHRM3* in schizophrenia. At a cellular level, mouse homologous *Hrh3* was significantly co-expressed with *Chrm3* among 5 out of 6 GABAergic neurons, including CCKC, MNC, PVC, CHC and ISC (all *P* values<1×10^-5^ and r^2^> 0.4; **Supplementary Figure 4d**). For mouse homologous *Chrna4*, it was also highly co-expressed with *Chrm3* among 3 GABAergic neurons, which are CCKC, MNC and ISC. In addition, both in THAL and PUTM, *HRH3* was significantly co-expressed with *CHRM3* (both *P* values<1×10^-10^ and r^2^>= 0.5). As our top SNP rs115455482 was an eQTL in THAL (weak association signal in PUTM), the unique co-expression between *CHRM3* and *HRH3* in the two brain tissues suggests that expression variation of *CHRM3* in THAL and PUTM may be also a risk factor for schizophrenia. Taken together, we show that *CHRM3* is strongly co-expressed with *GABRG2*, *HRH3*, and *CHRNA4*, which are risk genes involved in psychotic disorders, such as epilepsy and schizophrenia, indicating that the association between the genotype of *CHRM3* regulatory variant rs115455482 and cannabis-induced hallucinations is biologically meaningful.

**The Association of *CHRM3* Variants with Other Diseases**

To examine whether *CHRM3* has been reported in association with other diseases in previous GWASs, we searched in GRASP [8], a database containing summary statistics for published GWASs, and found that other *CHRM3* variants, but not our top SNPs (rs115455482 and rs74722579) which were not included in GRASP, have been reported to be associated or possibly associated with epilepsy, postoperative nausea and vomiting, autism, bone mineral content, crystallized intelligence, hypertension, information process speed (digit symbol), insulin disposition index, and myocardial infarction, sudden cardiac arrest in patients with coronary artery disease (among these previously published GWAS, all these variants’ *P* values<1×10^-4^, see **Supplementary Table S4**). Except for association observed with insulin disposition index and autism which were specifically reported to be associated with *CHRM3* variants in Hispanic and Admixed American populations, all *CHRM3* association variations were reported in European population.

In our GWAS of Ca-HL, two epilepsy associated variants, including rs1591887 and rs10925980, were nominally significant in our data, and the effect betas were all positive. We did not consider a correction for multiple testing for these 23 variants in **Supplementary Table S4**, as only 9 unique signals emerged when considering linkage disequilibrium structures among these variants, which can be observed based on their similar association *P* values. Therefore, these results should be considered exploratory. Furthermore, *CHRM3* variants were also marginally associated with postoperative nausea and vomiting (rs2355230 and rs12034521) and information processing speed (digit symbol) (rs1578180 and rs1072319).

**The Collaborative Study of the Genetics of Alcoholism (COGA)**

COGA recruited high risk families through alcohol dependent probands from inpatient and outpatient treatment facilities across 7 sites in the United States [9, 10]. The Institutional Review Boards at all sites approved this study and written consent was obtained from all participants. Comparison families were ascertained from a variety of sources (e.g., driver’s license records, dental clinics). All participants were interviewed using versions of the Semi-Structured Assessment for the Genetics of Alcoholism (SSAGA) which is reliable and valid [11, 12]. Genome-wide genotypic data and relevant phenotypic data were available for 7,595 individuals of European ancestry (EA) and 3,381 individuals of African ancestry (AA). The prevalence of lifetime cannabis use was 66% (EA) and 75.4% (AA) respectively.

Genotyping was performed using a variety of platforms, including the Illumina Human 1M array [13], Illumina OmniExpress 12V1 array [14] and the Illumina 2.5M array [15] ( Illumina, San Diego, CA), as well as on the Smokescreen (BioRelm, Walnut, CA) array. Multiple samples were genotyped on at least two different arrays with concordance >99%. Nonetheless, array type was included as a covariate in all analyses. A pruned set of 47,000 variants that were genotyped on all platforms and had minor allele frequencies (MAF) >10% in the combined samples, Hardy-Weinberg Equilibrium (HWE) p-values >0.001, missing rates<2%, and were not in linkage disequilibrium (LD, defined as R2<0.5) were used to assess reported pedigree structure using identity-by-descent calculations in PLINK [16]. Mendelian inconsistencies were detected in PEDCHECK [17] and relationship structures were corrected while genotype inconsistencies were set to missing. The same set of SNPs was used to calculate ancestral principal components in Eigenstrat [18]. Family ancestry was assigned based on the individual ancestry of the greatest proportion of family members. Imputation was performed using SHAPEIT2 [19] and then Minimac3 [20] with the 1000 Genomes (EUR and AFR, Phase 3, October 2014; build hg19) as the reference. Samples of differing genotype arrays were imputed separately to account for variability in content. Post-imputation quality control included exclusion of SNPs with R2<0.30 and inclusion of variants with genotype probabilities ≥0.90. Mendelian inconsistencies were further verified in Pedcheck [17] using hard-called imputed genotypes. Variants with missing rates less than 5%, MAF >=1% and HWE p values >0.000001 were used in analyses. All variants were mapped to NCBI GRCh37.

**Supplementary References**

1. Trabzuni, D., et al., *Widespread sex differences in gene expression and splicing in the adult human brain.* Nat Commun, 2013. **4**: p. 2771.

2. Paul, A., et al., *Transcriptional architecture of synaptic communication delineates gABAergic neuron identity.* Cell, 2017. **171**(3): p. 522-539 e20.

3. Schizophrenia Working Group of the Psychiatric Genomics, C., *Biological insights from 108 schizophrenia-associated genetic loci.* Nature, 2014. **511**(7510): p. 421-7.

4. Zhou, X. and M. Stephens, *Genome-wide efficient mixed-model analysis for association studies.* Nat Genet, 2012. **44**(7): p. 821-4.

5. Ward, L.D. and M. Kellis, *HaploReg v4: systematic mining of putative causal variants, cell types, regulators and target genes for human complex traits and disease.* Nucleic Acids Res, 2016. **44**(D1): p. D877-81.

6. Okamura, Y., et al., *COXPRESdb in 2015: coexpression database for animal species by DNA-microarray and RNAseq-based expression data with multiple quality assessment systems.* Nucleic Acids Res, 2015. **43**(D1): p. D82-D86.

7. Wang, J., et al., *WEB-based GEne SeT AnaLysis Toolkit (WebGestalt): update 2013.* Nucleic Acids Res, 2013. **41**(W1): p. W77-W83.

8. Eicher, J.D., et al., *GRASP v2.0: an update on the Genome-Wide Repository of Associations between SNPs and phenotypes.* Nucleic Acids Res, 2015. **43**(Database issue): p. D799-804.

9. Edenberg, H.J., *The collaborative study on the genetics of alcoholism: an update.* Alcohol Res Health, 2002. **26**(3): p. 214-8.

10. Reich, T., et al., *Genome-wide search for genes affecting the risk for alcohol dependence.* Am J Med Genet, 1998. **81**(3): p. 207-215.

11. Bucholz, K.K., et al., *A New, Semistructured Psychiatric Interview for Use in Genetic-Linkage Studies - a Report on the Reliability of the Ssaga.* J Stud Alcohol, 1994. **55**(2): p. 149-158.

12. Hesselbrock, M., et al., *A validity study of the SSAGA - a comparison with the SCAN.* Addiction, 1999. **94**(9): p. 1361-1370.

13. Edenberg, H.J., et al., *Genome-Wide Association Study of Alcohol Dependence Implicates a Region on Chromosome 11.* Alcohol Clin Exp Res, 2010. **34**(5): p. 840-852.

14. Wang, J.C., et al., *A genome-wide association study of alcohol-dependence symptom counts in extended pedigrees identifies C15orf53.* Mol Psychiatry, 2013. **18**(11): p. 1218-24.

15. Meyers, J.L., et al., *An endophenotype approach to the genetics of alcohol dependence: a genome wide association study of fast beta EEG in families of African ancestry.* Molecular Psychiatry, 2017. **22**(12): p. 1767-1775.

16. Purcell, S., et al., *PLINK: A tool set for whole-genome association and population-based linkage analyses.* Am J Hum Genet, 2007. **81**(3): p. 559-575.

17. O'Connell, J.R. and D.E. Weeks, *PedCheck: A program for identification of genotype incompatibilities in linkage analysis.* Am J Hum Genet, 1998. **63**(1): p. 259-266.

18. Price, A.L., et al., *Principal components analysis corrects for stratification in genome-wide association studies.* Nat Genet, 2006. **38**(8): p. 904-909.

19. Delaneau, O., J.F. Zagury, and J. Marchini, *Improved whole-chromosome phasing for disease and population genetic studies.* Nature Methods, 2013. **10**(1): p. 5-6.

20. Das, S., et al., *Next-generation genotype imputation service and methods.* Nat Genet, 2016. **48**(10): p. 1284-1287.
